# Precise Definition of Porcine Hippocampal Cornu Ammonis 2: High Histoarchitectural Similarity to Humans but Unequal Sensitivity to Hypoxia

**DOI:** 10.3390/biomedicines12081896

**Published:** 2024-08-19

**Authors:** Miriam Renz, Pascal Siegert, Katja Mohnke, Robert Ruemmler, Katrin Frauenknecht, Clemens Sommer, Anja Harder

**Affiliations:** 1 Department of Anesthesiology, University Medical Center of the Johannes Gutenberg University Mainz, 55131 Mainz, Germany; 2 Institute of Neuropathology, University Medical Center of the Johannes Gutenberg University Mainz, 55131 Mainz, Germany; 3 Department of Cancer Research (DoCR), Luxembourg Center of Neuropathology (LCNP), Luxembourg Institute of Health (LIH), 1210 Luxembourg, Luxembourg; 4 Research Center for Immunotherapy (FZI), University Medical Center of the Johannes Gutenberg University Mainz, 55131 Mainz, Germany

**Keywords:** hippocampus, cornu ammonis, ischemia, pig studies, animals, cardiac arrest studies, hypoxic–ischemic brain injury

## Abstract

Experimental animal studies of hypoxic–ischemic injury of the hippocampus of pigs are limited due to the unprecise definition of hippocampal subfields, cornu ammonis 1 to 4, compared to humans. Given that the pig model closely mirrors human physiology and serves as an important model for critical care research, a more precise description is necessary to draw valid conclusions applicable to human diseases. In our study, we were able to precisely define the CA2 and its adjacent regions in a domestic pig model by arginine vasopressin receptor 1B (AVPR1B) and calbindin-D28K like (CaBP-Li) expression patterns. Our findings demonstrate that the histoarchitecture of the porcine cornu ammonis subfields closely resembles that of the human hippocampus. Notably, we identified unusually strong neuronal damage in regions of the pig hippocampus following global ischemia, which are typically not susceptible to hypoxic–ischemic damage in humans.

## 1. Introduction

Traditionally, studying ischemic neuronal damage after hypoxia focusses on hippocampal subfields. The hippocampus has a highly plastic structure, and its functionally different subfields show unequal vulnerability to hypoxia. In humans, the cornu ammonis 1 (CA1) is most susceptible to ischemia, whereas CA3 is less susceptible. Many different factors are discussed to explain the higher sensitivity of CA1 neurons to oxygen and glucose deprivation by ischemia [1]. We intended to measure the extent of ischemic neuronal damage within the established hypoxia-sensitive area CA1 in pigs. However, a comprehensive literature search for an appropriate anatomical definition of CA3, CA2, and CA1 in pigs left us unsatisfied. Therefore, we wanted to precisely define the CA subfields in pigs in order to establish the pig as a hypoxia/ischemia experimental model.

## 2. Materials and Methods

**Figure 1:**
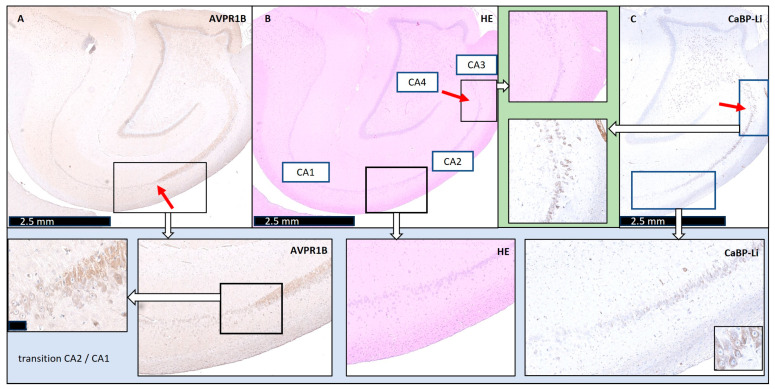
Definition of the pigs’ normal cornu ammonis CA2 and transitions to adjacent regions in the porcine hippocampus. (**Upper picture row**) (all scale bars: 2.5 mm): (**A**) **AVPR1B:** The left image shows arginine vasopressin receptor 1B (AVPR1B) staining. The red arrow highlights the CA1-CA2 transition. (**B**) **HE:** The middle image shows hematoxylin–eosin (HE) staining, with CA1 to CA4 labeled. (**C**) **CaBP-Li:** The right image presents calbindin-D28K-like (CaBP-Li) staining. The red arrows in the HE and CaBP-Li images show the CA2-CA3 transition, which is also shown enlarged in the upper row, with a green background (cutout). (**Lower picture row**) The lower row shows cutouts of the images of the AVPR1B, HE, and CaBP-Li staining to highlight the appearance of the CA1-CA2 transition. Scale bar of the AVRBP1 cutout: 50 µM.

**Figure 2:**
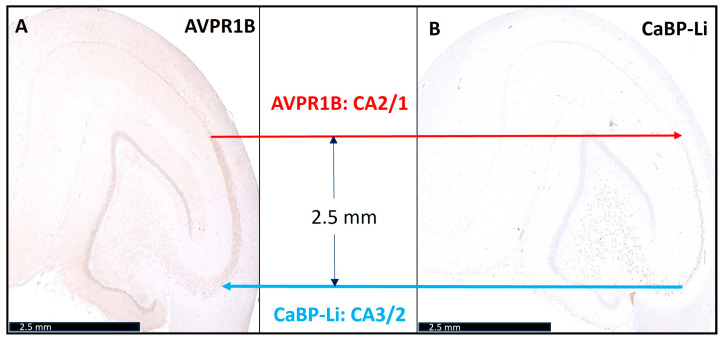
Precise definition of the pigs’ normal length of cornu ammonis (CA2). The left image (**A**) shows arginine vasopressin receptor 1B (AVPR1B) staining. The red arrow highlights the CA1-CA2 transition. The right image (**B**) shows calbindin-D28K-like (CaBP-Li) staining. The blue arrow highlights the CA2-CA3 transition. The length of CA2 region is approximately 2.5 mm. Scale bars: 2.5 mm.

We combined anatomical and immunohistochemical investigations to define porcine CA2 applying recently reported specific protein expression patterns of the different hippocampal subfields. We used landrace pigs weighting 29–34 kg and who were 12–16 weeks old (approval no. G21-1-080, State and Institutional Animal Care Committee Rhineland Palatine, Germany). The animals received an anxiolytic regimen at their local farm before transportation to our Animal Research Facility. The pigs were anesthetized after receiving standard monitoring. The airway was secured, and they were mechanically ventilated. For the definition of CA2, four pigs were euthanized after the named procedures, and the hippocampus was examined as described below (Figure 1 and Figure 2). To assess the extent of ischemic neuronal damage, the pigs underwent an intervention—cardiopulmonary resuscitation (CPR) [2,3]—potentially resulting in ischemic neuronal damage. Therefore, the aforementioned anesthetized pigs were instrumented and equipped with measuring tools, and ventricular fibrillation was induced. The no-flow time was 8 min, followed by 8 min of basic life support and then advanced life support (low-flow time), using different ventilation strategies [2]. No- and low-flow times as well as the different ventilation strategies led to variable oxygenation during CPR [3]. If a return of spontaneous circulation (ROSC) was reached, the pigs were monitored for 22 h and then euthanized.

After euthanasia, the brain was removed immediately and fixed in a 4% formaldehyde solution. After 4 weeks, the brain was sliced in order to display the hippocampus as the region of interest (ROI). To correctly identify the ROI, a pig brain atlas was used [4]. The paraffin-embedded tissue was cut into 2–5 μm thick sections on a microtome, mounted on glass slides and stained with hematoxylin–eosin (HE) as well as Nissl staining, silver staining, and immunohistochemical staining. First, we analyzed the calcium-binding protein calbindin-D28K-like (CaBP-Li) (Supplementary Materials), since human CA2 exhibits a specific expression of CaBP-Li [5]. Secondly, we analyzed a vasopressin receptor highly expressed in CA2 [5] by applying an antibody against arginine vasopressin receptor 1B (AVPR1B) Supplementary Materials), as shown for other species.

## 3. Results

When analyzing the two immunohistochemical stains in combination with conventional histochemistry, the porcine CA2 could be clearly delineated from CA1 and CA3. During the transition from CA2 to CA1, an obvious broadening of the neuronal band could be recognized, similar to the well-known human architecture (Figure 1). In addition, we were also able to confirm the findings of Bartesaghi and Ravasi in guinea pigs [6]. We defined the length of CA2 to be approximately 2.5 mm ± 0.02 mm (*n* = 4) (Figure 2). Surprisingly, after precisely defining the CA subfields, it appeared that pig neurons of CA3 (which underwent cardiac arrest, CPR, and ROSC, as described in the Methods Section) showed signs of hypoxic–ischemic brain injury (HIBI).

## 4. Discussion

In our analyses, the different CA subfields in the pig brain were precisely defined. To define CA2, we used an antibody against AVPR1B, which is highly expressed in CA2 in other species. We also used an antibody against CaBP-Li, as human CA2 has a specific expression of CaBP-Li [5]. Interestingly, pyramidal CA2 neurons in monkeys show a higher reactivity for calcium-binding proteins [7]. Histologically, CA2 neurons are not as densely packed as in CA1, but they are morphologically relatively like those in CA3, making them difficult to distinguish on conventional slides. Nevertheless, CA2 can be distinguished by unique molecular and pharmacological profiles. Compared to CA1 and CA3, Dudek et al. identified specific properties such as distinct molecular profiles and connectivity, modulation by caffeine, oxytocin and vasopressin, and a high expression of the vasopressin receptor [5]. Interestingly, sequence-based genome data show that guinea pigs exhibit a contradictory monophyly compared to other laboratory rodents such as rats and mice [8]. Previous studies in guinea pigs investigated the anatomical structures of CA2 by conventional staining and defined neuronal population according to the criteria in the mouse hippocampus [6]. Here, the CA2 area was relatively short (average 300 µm) compared to humans and monkeys, but larger than in other rodents. It contained pyramidal neurons with one or two apical dendrites, although some neurons were CA1-like (monoapical) and CA3-like. They observed that CA2 exhibited four different neuron types according to the number of dendrites, determining different synaptic contacts [6]. Interestingly, CA2 had already been described as a mixed zone (“Mischzone”) as early as in 1908 and was defined by Braak in detail for humans [9]. These data underline that it is not possible to directly define CA2 between different species by conventional approaches.

We now accurately defined the porcine CA2. In former porcine cardiac arrest models, some studies did not precisely distinguish the hippocampal regions. Such approaches did not allow to distinguish hypoxia-sensitive and -resistant neurons, although they used a detailed scoring system [10]. Our definition of the porcine CA subfields supports the use of pigs as a suitable model for spatial expression analyses and investigating the effects of different experimental conditions, such as hypoxia/ischemia. We found that CA3 neurons in pigs are highly vulnerable to hypoxia, which contrasts with the assumed human condition. We did not perform the same approach applied to human brains, as there are already very precise studies characterizing this hippocampal region in detail [11]. Also, as our goal was to accurately determine the porcine CA regions, we did not intend to determine the extent of ischemia or quantify ischemia more precisely in the porcine CA regions. We were surprised to find ischemia in CA3, as it is contrary to the human situation. This finding suggests that, despite the anatomical substructure being extremely similar, pigs may have different plasticity and functional circuits compared to humans. We are going to investigate the exact extent and distribution of ischemia in the porcine CA regions in a separate study.

## 5. Conclusions

In summary, immunochemical staining for AVPR1B and CaBP-Li, along with conventional histochemistry, precisely defined porcine CA2, distinguishing it from adjacent regions and showing a strong resemblance to human architecture. These findings will enhance future porcine studies on hippocampal areas, for example, when investigating neuronal hypoxia (HIBI). Additionally, CA3 neurons showed vulnerability to hypoxia, which we will further investigate using our cardiac arrest pig model.

## Data Availability

All data analyzed for this study are provided in the manuscript.

## Supplementary Materials

The following supporting information can be downloaded at https://www.mdpi.com/article/10.3390/biomedicines12081896/s1: Text S1: Immunohistochemical staining protocols for antibodies against calbindin-D28K like (CaBP-Li) and arginine vasopressin receptor 1B (AVPR1B).
