# Supplementary material for: Precise Definition of Porcine Hippocampal Cornu Ammonis 2: High Histoarchitectural Similarity to Humans but Unequal Sensitivity to Hypoxia
Source: Biomedicines. 2024 Aug 19;12(8):1896. doi: 10.3390/biomedicines12081896 (PMC11351859; doi:10.3390/biomedicines12081896)
Supplement: Supplementary file 1 [file biomedicines-12-01896-s001.zip › biomedicines-3096126-supplementary.pdf]

Supplementary Materials

Immunohistochemical staining protocols for antibodies against calbindin-D28K like (CaBP-Li) and arginine vasopressin receptor 1B (AVPR1B)

Slices of porcine hippocampal FFPE brain tissue samples were deparaffinized. For pre-treatment the tissue was cooked for 35 minutes in citrate buffer (EnVision FLEX Target Retrieval Solution low ph, DAKO) at pH 6.0 and cooled under running water thereafter.

To demonstrate CaBP-Li we used a monoclonal biotinylated anti-mouse Calbindin D28K (D-4) antibody (Santa Cruz Biotechnology, USA) at a dilution of 1:50. To stain AVPR1B a polyclonal anti-rabbit AVPR 1B antibody (Vector Laboratories, USA) at a dilution of 1:50 was applied.

After treatment with 3 % H_2O_2 (ROTH) in buffered methanol (MERCK) for 30 minutes, incubation in normal goat serum (1:50) over night, and incubation with a biotinylated anti-mouse antibody (1:50) for 1 h, a Vectastain Standard ABC Kit (Vector Laboratories, USA) as well as a DAB Ultravision detection system (Thermo Fischer Scientific, Germany) were used for CaBP-Li staining.

To stain AVPR1B, endogenous biotin was blocked using an avidin/biotin blocking kit (Vector Laboratories, USA). Then, samples were incubated with the polyclonal antibody overnight, and with the secondary anti-rabbit antibody of the Vectastain kit thereafter. For following procedures, the Vectastain Standard ABC Kit (Vector Laboratories, USA) and the DAB Ultravision detection system (Thermo Fischer Scientific, Germany) were used as well.

Final counterstaining was performed with hematoxylin (EPREDIA, No 7211). Stained sections were mounted with Cytolseal XYL Mounting Medium (Richard-Allan Scientific, USA).
